# Exploring the cellular surface polysaccharide and root nodule symbiosis characteristics of the *rpoN* mutants of *Bradyrhizobium* sp. DOA9 using synchrotron-based Fourier transform infrared microspectroscopy in conjunction with X-ray absorption spectroscopy

**DOI:** 10.1128/spectrum.01947-23

**Published:** 2023-09-08

**Authors:** Jenjira Wongdee, Pongdet Piromyou, Pongpan Songwattana, Teerana Greetatorn, Nantakorn Boonkerd, Neung Teaumroong, Eric Giraud, Djamel Gully, Nico Nouwen, Worawikunya Kiatponglarp, Waraporn Tanthanuch, Panlada Tittabutr

**Affiliations:** 1 Institute of Research and Development, Suranaree University of Technology, Muang, Nakhon Ratchasima, Thailand; 2 School of Biotechnology, Institute of Agricultural Technology, Suranaree University of Technology, Muang, Nakhon Ratchasima, Thailand; 3 IRD, IRD/CIRAD/INRAE/Université de Montpellier/SupAgro, Plant Health Institute of Montpellier, UMR-PHIM, Montpellier, France; 4 Thai wah public company limited @CU innovation hub, Faculty of Science, Chulalongkorn University, Bangkok, Thailand; 5 Synchrotron Light Research Institute (Public Organization), Muang, Nakhon Ratchasima, Thailand; Weizmann Institute of Science, Rehovot, Israel

**Keywords:** sigma factor, RpoN, *Bradyrhizobium*, FTIR and XAS, nodulation, cellular surface polysaccharide, colony formation

## Abstract

The functional significance of *rpoN* genes that encode two sigma factors in the *Bradyrhizobium* sp. strain DOA9 has been reported to affect colony formation, root nodulation characteristics, and symbiotic interactions with *Aeschynomene americana. rpoN* mutant strains are defective in cellular surface polysaccharide (CSP) production compared with the wild-type (WT) strain, and they accordingly exhibit smaller colonies and diminished symbiotic effectiveness. To gain deeper insights into the changes in CSP composition and the nodules of *rpoN* mutants, we employed synchrotron-based Fourier transform infrared (SR-FTIR) microspectroscopy and X-ray absorption spectroscopy. FTIR analysis of the CSP revealed the absence of specific components in the *rpoN* mutants, including lipids, carboxylic groups, polysaccharide-pyranose rings, and β-galactopyranosyl residues. Nodules formed by DOA9WT exhibited a uniform distribution of lipids, proteins, and carbohydrates; mutant strains, particularly DOA9∆*rpoNp*:Ω*rpoNc*, exhibited decreased distribution uniformity and a lower concentration of C=O groups. Furthermore, Fe K-edge X-ray absorption near-edge structure and extended X-ray absorption fine structure analyses revealed deficiencies in the nitrogenase enzyme in the nodules of DOA9∆*rpoNc* and DOA9∆*rpoNp*:Ω*rpoNc* mutants; nodules from DOA9WT and DOA9∆*rpoNp* exhibited both leghemoglobin and the nitrogenase enzyme.

**IMPORTANCE:**

This work provides valuable insights into how two *rpoN* genes affect the composition of cellular surface polysaccharides (CSPs) in *Bradyrhizobium* sp., which subsequently dictates root nodule chemical characteristics and nitrogenase production. We used advanced synchrotron methods, including synchrotron-based Fourier transform infrared (SR-FTIR) microspectroscopy and X-ray absorption spectroscopy (XAS), for the first time in this field to analyze CSP components and reveal the biochemical changes occurring within nodules. These cutting-edge techniques confer significant advantages by providing detailed molecular information, enabling the identification of specific functional groups, chemical bonds, and biomolecule changes. This research not only contributes to our understanding of plant-microbe interactions but also establishes a foundation for future investigations and potential applications in this field. The combined use of the synchrotron-based FTIR and XAS techniques represents a significant advancement in facilitating a comprehensive exploration of bacterial CSPs and their implications in plant-microbe interactions.

## INTRODUCTION

Symbiotic rhizobia contribute to host legumes’ growth, development, and yield by fixing active nitrogenase from atmospheric N_2_ to N compounds. Nitrogenase is a structurally conserved two-component enzyme complex responsible for nitrogen fixation in nitrogen-fixing bacteria (1). The expression of nitrogen-fixing genes is regulated by hierarchically organized regulatory genes, allowing the bacteria to respond to optimal environmental conditions (2). In most nitrogen-fixing bacteria, the NifA-RpoN regulatory cascade regulates the transcription of *nif* genes, and their expression occurs when both regulatory NifA and RpoN recognize the upstream activating sequence at the upstream region of the transcriptional start site (3) on the regulated gene. However, the efficiency of promoting host plant growth phenotypes depends on nodulation signaling, bacterial metabolism, and activity in nitrogen fixation.

In addition to species-specific interaction signaling that occurs during the initiation of the nodulation process, other factors produced by specific microorganisms may also be involved, such as components in bacterial cellular surface polysaccharides (CSPs), including cyclic β-glucans (CβGs), exopolysaccharides (EPSs), lipopolysaccharides (LPSs), capsular polysaccharides (KPSs or K-antigens), and biofilm formation (4 – 7). Several research studies have reported the role of bacterial CSPs in symbiotic infection and nodulation with specific host plants. The CSPs, slightly attached to the surface of the outer bacterial membrane, are considered essential for establishing nitrogen-fixing symbiosis in developing legumes (5, 6, 8). Cyclic glucans, found in the periplasmic compartment of *Bradyrhizobium japonicum*, are sometimes also secreted for root attachment during plant infection and hypo-osmotic adaptation (9). The CSP structure and biosynthesis pathway have been reported differently in *Sinorhizobium meliloti* and *Rhizobium favelukesii* LPU83 and are also involved in symbiotic colonization with determinate nodule meristems of alfalfa plants (6, 10).

*Bradyrhizobium* sp. strain DOA9 is a model for nitrogen-fixing bacteria that effectively promotes nitrogen fixation under symbiosis with *Aeschynomene americana* and free-living conditions (11). Genome analysis revealed that DOA9 contains one chromosome (c) and one symbiotic plasmid (p), both of which carry *nif* genes, including the nitrogenase structural genes, *nifH*, *nifD*, and *nifK*, and nitrogenase regulatory genes, *nifA* and *rpoN* (12). Our recent study demonstrated that deletion mutations of the nitrogenase regulatory *rpoN* genes in DOA9 cause a reduction in CSP production under free-living conditions (13). All *rpoN* mutant strains exhibited smaller colonies with different CSP characteristics than the wild type (WT), which is related to their lesser effectiveness in producing root nodules and plant growth phenotypes. Interestingly, a reduction in nitrogen fixation activity with the appearance of small and less red-colored nodules was found in *A. americana* inoculated with *rpoN* mutant strains. Although the CSP production and symbiotic nitrogen fixation efficiency of these mutants have been investigated by analyzing the weight of dried CSP, nodule appearance under microscopes, and nitrogenase activity assays, respectively (13), these methods could only represent the physiological function of the two RpoN in DOA9 that affected CSP production, nitrogen fixation, and nodule phenotypes. Therefore, a deeper understanding of the functional components in the CSP structure of cell growth under free-living conditions and the biochemical changes in nodules formed by the *rpoN* mutants requires advanced analysis technology.

Various microspectroscopic methods, including vibrational infrared radiation (IR) and X-ray, can be used to analyze the composition of complex chemical compounds and the biochemical changes at the cellular level (14 – 16). Fourier transform infrared (FTIR) spectroscopy is a powerful analytical technique that measures a sample’s absorption or transmission of infrared light (17). To analyze exopolysaccharides, FTIR spectroscopy can identify specific types of sugar moieties present in the polysaccharide structure (5, 18). Plant-microbe interactions can also be studied using FTIR spectroscopy (19, 20). Using the spectral patterns produced by the analysis, it is possible to identify changes in the chemical composition of the samples, such as the presence of specific functional groups or the formation of new chemical bonds (17).

Furthermore, advanced X-ray absorption spectroscopy (XAS) analysis techniques, such as energy-dispersive X-ray spectroscopy (EDS), X-ray absorption near-edge structure (XANES), and extended X-ray absorption fine structure (EXAFS), are used to determine the chemical components and chemical formations in biomolecule structure (15, 21). This information leads to an understanding of the physical and mechanical properties of the plant cell wall (15). Microbes are essential for the health of plants, as they can help plants acquire nutrients, protect them from pathogens, and enhance their resistance to environmental stress (2). XAS can analyze how microbes interact with plant tissues and identify the chemical changes due to these interactions. This information can be used to develop new strategies for improving legume nodulation and nitrogen fixation processes, further increasing crop yields (22, 23).

**Fig 1:**
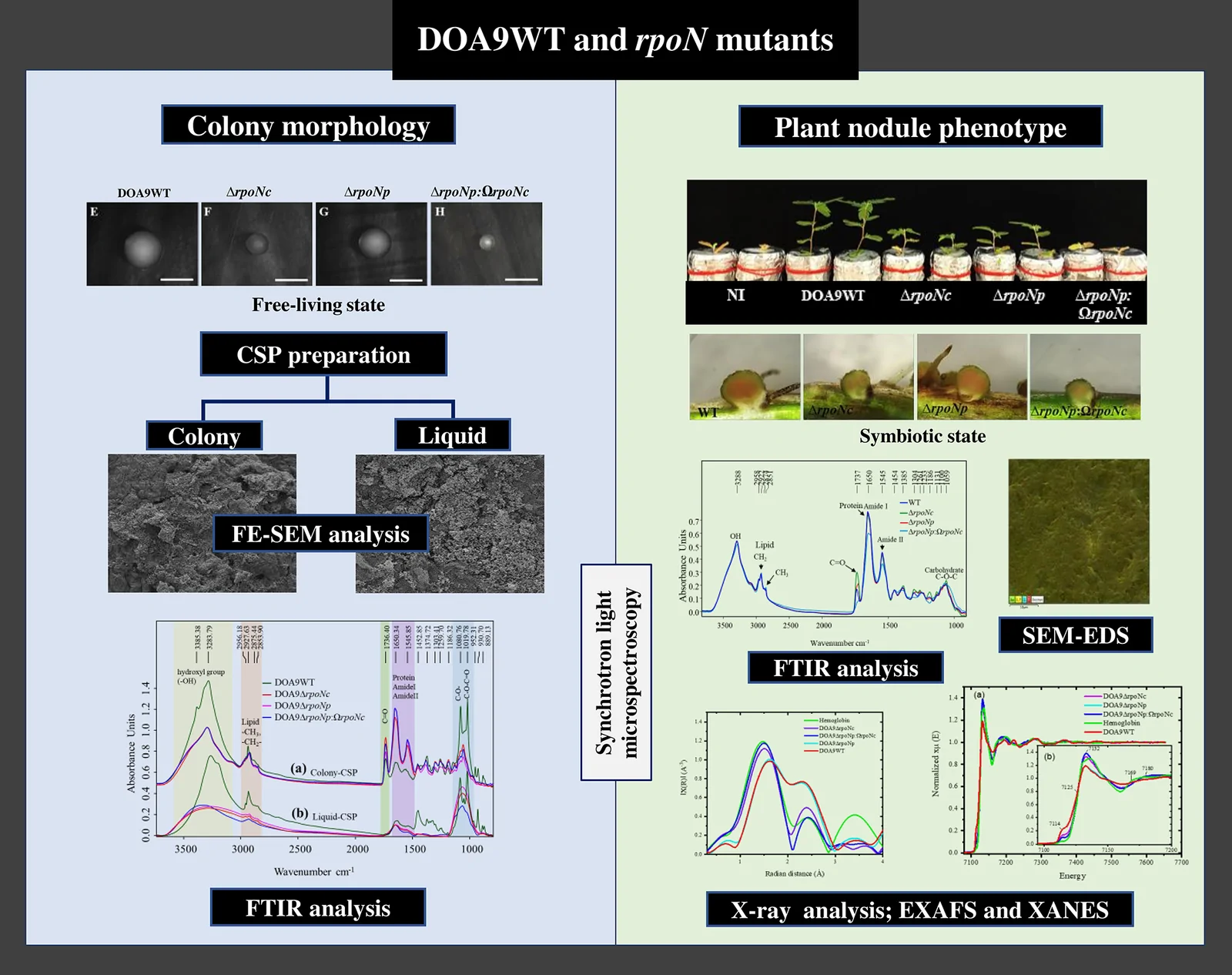
The experimental schematic is planned for the analysis of two DOA9RpoN effects on colony morphology and plant nodule phenotype using synchrotron-based light microspectroscopy, including FTIR and X-ray analyses. To analyze the polysaccharide structure components of CSP produced from DOA9 derivatives, FE-SEM and FTIR were applied (left). FTIR and X-ray spectroscopy (right) were used to examine the biochemical composition of root nodules.

Microspectroscopy provides unique molecular chemical information that facilitates the identification of chemical components and structure formations of biomolecules or materials and has become increasingly relevant in the study of plant-microbe interactions (22, 24). To determine the biochemical information involved in the nitrogen fixation activity of nodules and with the different CSP production phenotypes of DOA9WT and its *rpoN* mutant derivatives, this study used (i) FTIR microspectroscopy to identify changes in the chemical functional groups within the CSP produced by DOA9 and its derivatives and in the root nodules, and (ii) XAS analysis (EDS, XANES, and EXAFS) to detect biochemical changes in the nodules of *A. americana* inoculated with DOA9WT and *rpoN* mutant strains (see the experimental schematic in Fig. 1). This provides detailed information on the molecular structure of the samples, which can be used to gain insight into their properties and potential applications during interactions between plants and microbes.

## RESULTS

### The CSP microstructures of *Bradyrhizobium* sp. DOA9 and DOA9*rpoN* mutant strains

**Fig 2:**
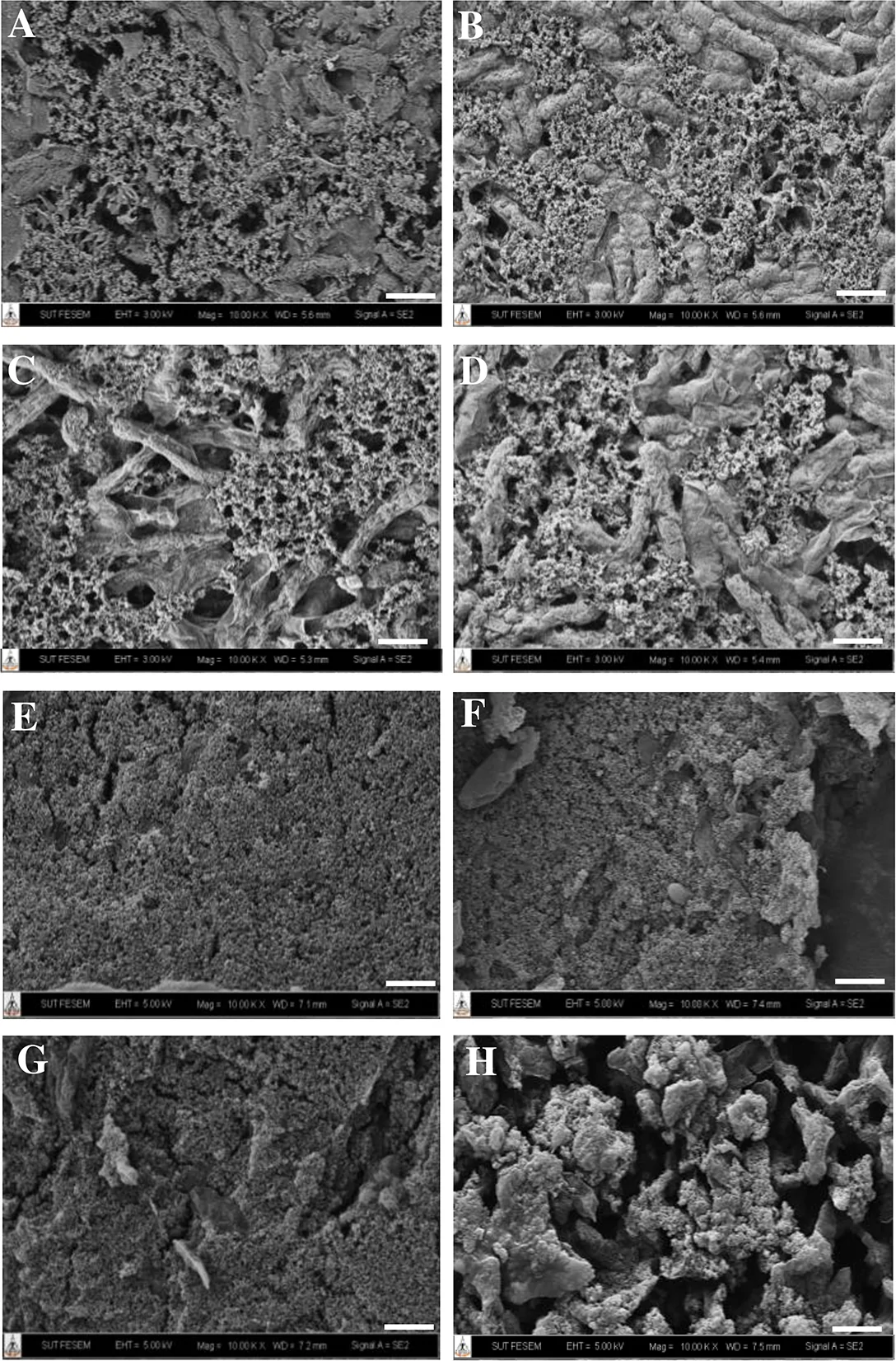
CSP structure morphology was observed under FE-SEM; FE-SEM image of colony-CSP (**A to D**) and liquid-CSP (**E to H**), structure morphology of DOA9WT (**A and E**), DOA9∆*rpoNc* (**B and F**), DOA9∆*rpoNp* (**C and G**), and DOA9∆*rpoNp*:Ω*rpoNc* (**D and H**). Bar on the FE-SEM image stands for 1 µm.

**Fig 3:**
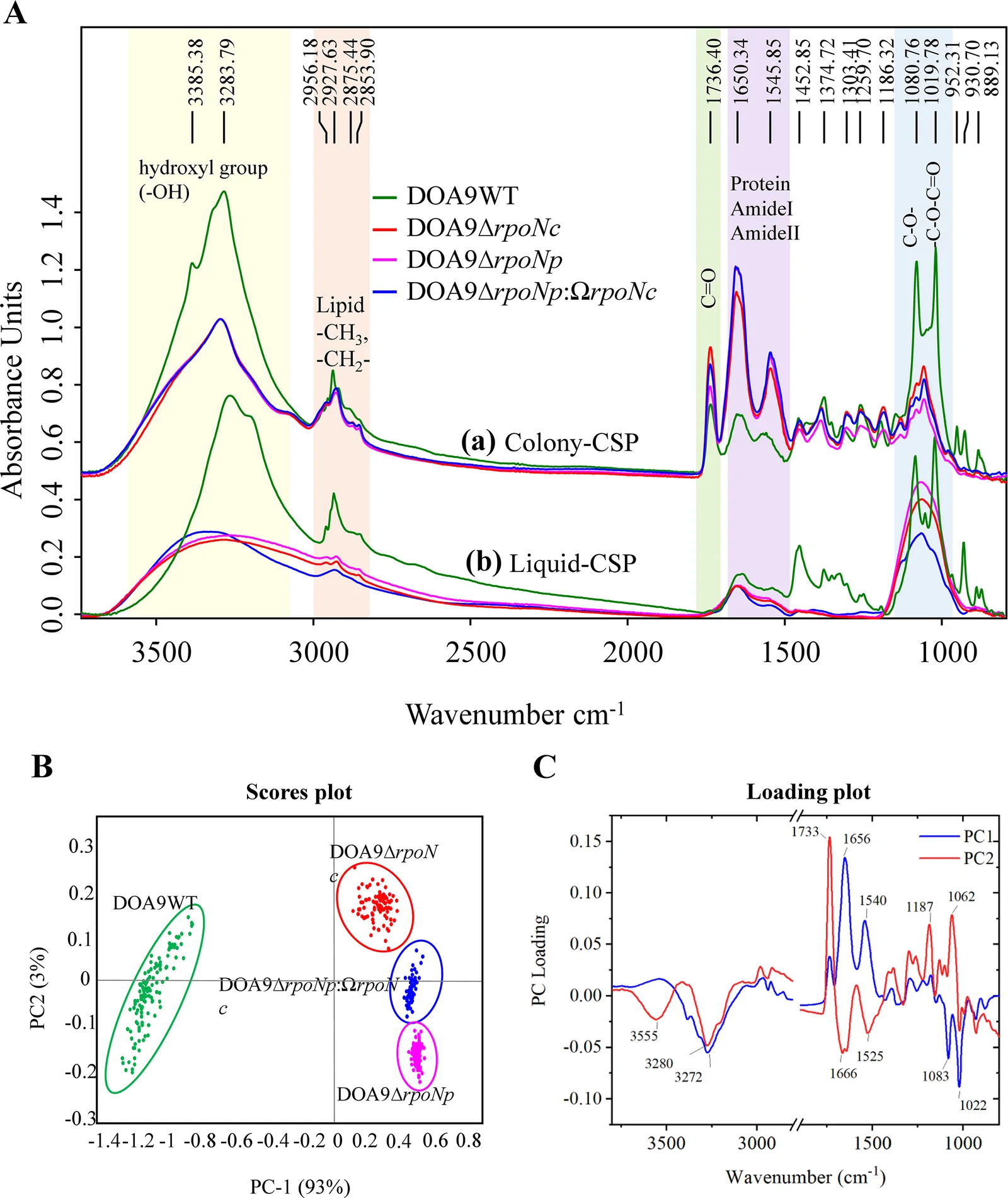
Average FTIR spectra (**A**) of CSP extracted from colony-CSP (a) and liquid-CSP (b) of *Bradyrhizobium* sp. strain DOA9WT, DOA9∆*rpoNc*, DOA9∆*rpoNp*, and DOA9∆*rpoNp*:Ω*rpoNc*. Scores plot of PCA analysis between PC1 and PC2 of colony-CSP (**B**). Loading plot between PC1 and PC2 of colony-CSP (**C**).

To confirm the effect of the RpoN mutation on the colony size of strain DOA9 (13), single colonies from both the DOA9WT and DOA9∆*rpoN* mutant strains were examined on a yeast extract-mannitol (YEM) agar plate. The mutant strains were smaller (Fig. S1A through D in the supplementary material). The dried CSP powder extracted from different cultures (solid and liquid medium; see the Materials and Methods section) of DOA9WT and DOA9∆*rpoN* mutants was observed by a field emission scanning electron microscope (FE-SEM). The FE-SEM images showed rough and irregular surfaces for all colony-CSP (Fig. 2A through D) and liquid-CSP (Fig. 2E through H). However, the morphology of the liquid-CSP obtained from the DOA9WT and DOA9∆*rpoNp* strains (Fig. 2E and G) was more compact than that of the DOA9∆*rpoNc* and DOA9∆*rpoNp*:Ω*rpoNc* strains (Fig. 2F and H). Moreover, the micromorphology of CSP isolated from the liquid of DOA9*∆rpoNc* and DOA9∆*rpoNp*:Ω*rpoNc* was slightly smoother than the other samples (Fig. 2F and H). On the other hand, the colony-CSP image contained bacterial cells (Fig. 2A through D). This finding could possibly be attributed to the detection of the amide I and amide II bands in FTIR analysis, which indicates bacterial biomolecules [Fig. 3A(a)].

### CSP analysis using synchrotron FTIR microspectroscopy

The FTIR spectral features of both colony-CSP and liquid-CSP of the DOA9WT were significantly different from those of the mutant strains (DOA9∆*rpoNc*, DOA9∆*rpoNp,* and DOA9∆*rpoNp*:Ω*rpoNc*) [Fig. 3A(a) and (b)]. The functional groups present in CSP can be identified by the characteristic FTIR peaks. The stretching frequency range of 3,000–3,700 cm^−1^, which corresponds to the vibrations of carbohydrate hydroxyl (-OH) group (25, 26), was the broadest and most intense in DOA9WT. This was also correlated with the highest yield production of CSP in this strain. Furthermore, the methyl (CH_3_) and methylene (CH_2_) groups of lipopolysaccharides detected at 2,927 cm^−1^ and 2,958 cm^−1^, respectively (27), exhibited the most intense and distinct stretching modes in wild-type CSP. Conversely, in all mutant strain CSPs, these stretching modes appeared as broad peaks [Fig. 3A(a) and (b)]. The spectral feature and intensity of the vibration between 1,180 and 940 cm^−1^, which corresponds to the C–O and C–C stretching vibrations of the polysaccharides-pyranose ring, were similar in the colony-CSP of all mutant strains. In contrast, a distinct C–O stretching vibration peak was detected at 1,080 cm^−1^ and 1,019 cm^−1^ in both the colony-CSP and liquid-CSP of the wild-type strain [Fig. 3A(a) and (b)], which supports the presence of distinct CSP structures.

Interestingly, the liquid-CSP of DOA9WT showed a distinct high intensity of lipid bands at 3,000–2,800 cm^−1^ and a C–H band at around 1,452 cm^−1^, which belong to glucuronic acid (28). Furthermore, the strong IR bands at 930, 889, and 874 cm^−1^ [Fig. 3A(a) and (b)], which correspond to the anomeric CH of β-galactopyranosyl residues (29), were detected in DOA9WT. In contrast, these peaks were absent in all mutant strains, implying that the mutations caused the alteration of both the LPS molecule production and the repetitive glycan polymer attachment to the core domain.

The presence of bacterial cells within the colony-CSP (Fig. 2A through D) contributed to the high intensity of the C=O and N–H signals (between 1,500 cm^−1^ and 1,700 cm^−1^) observed in the amide I and amide II bands of proteins [Fig. 3A(a)]. On the other hand, the intensity of these signals was lower in the liquid-CSP [Fig. 3A(b)]. These findings suggest that the *Bradyrhizobium* sp. strain DOA9 produces a higher density or longer chains of CSP in solid cultures when compared to liquid cultures, which affects the extraction of CSP due to the complex structure of CSP that holds bacteria inside. The presence of phospholipid head groups (C=O stretching at 1,740 cm^−1^) in the colony-CSP of all strains supports the presence of bacterial cells that contribute to the lipid bilayer of bacterial cell membranes, which is not observed in liquid-CSP [Fig. 3A(b)].

Principal component analysis (PCA) of FTIR spectra of CSP was performed to reduce the dimensionality of a data set while retaining as much of the original information as possible, then identify the most significant features or patterns in the spectra for group classification. The original data were transformed into a new set of variables called principal components (PCs). All spectral data without the biological components in colony-CSP from functional groups of amide I and II suggested the similarity of all detected liquid-CSP spectra. The PCs analysis showed that the best clustering of the different classes of colony-CSP was achieved using PC1 versus PC2 (Fig. 3B). The negative PC1 (representing 93% of the scores) was used to separate DOA9WT from the mutants. This negative PC1 score was associated with the lipid region (3,272 cm^−1^) and the carbohydrate region (1,083 cm^−1^ and 1,022 cm^−1^), as indicated by the negative values of the PC1 loadings plot. DOA9∆*rpoNc* can be distinguished from DOA9∆*rpoNp* by the positive PC2 (3% scores plot), which is associated with the lipid head group (1,733 cm^−1^) and carbohydrate region (1,187 cm^−1^ and 1,062 cm^−1^) of positive PC2 loading (Fig. 3C).

Figure S2 (in the supplement material) shows the interpretation of colony-CSP by FTIR peaks, which include –OH, CH_2_, and CH_3_ (lipid), C=O, –C–O–C (carbohydrate), and –COOH (protein). This result demonstrates the percentage integral area of each characteristic FTIR peak, indicating that the pattern ratio of biological molecules in the CSP-wild-type strain significantly differs from that of the mutant strains. This finding indicates that the mutation in the *rpoN* gene, present in either the chromosome, plasmid, or both locations of *Bradyrhizobium* sp. strain DOA9, can have an impact on the chemical structure of the CSP.

### Analysis of biochemical composition in plant nodule using SR-FTIR microspectroscopy

**Fig 4:**
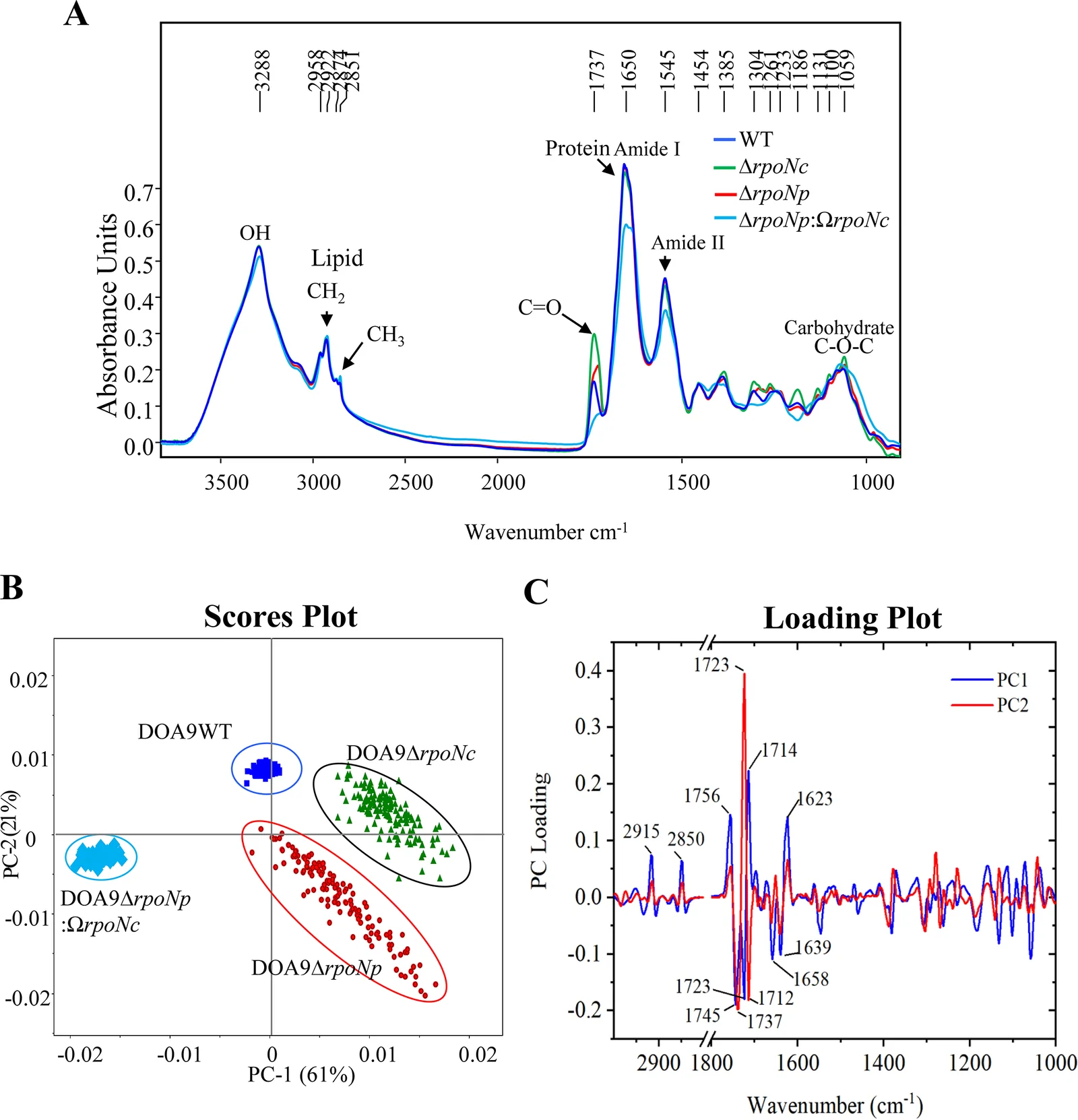
Average FTIR spectra of nodules from *A. americana* that were elicited with different strains of *Bradyrhizobium* sp. DOA9WT, DOA9∆*rpoNc*, DOA9∆*rpoNp,* and DOA9∆*rpoNp*:Ω*rpoNc* after 20 days of inoculation under light room condition (**A**). Scores plot of PCA analysis between PC1 and PC2 (**B**). Loading plot of PC1 and PC2 (**C**).

**Fig 5:**
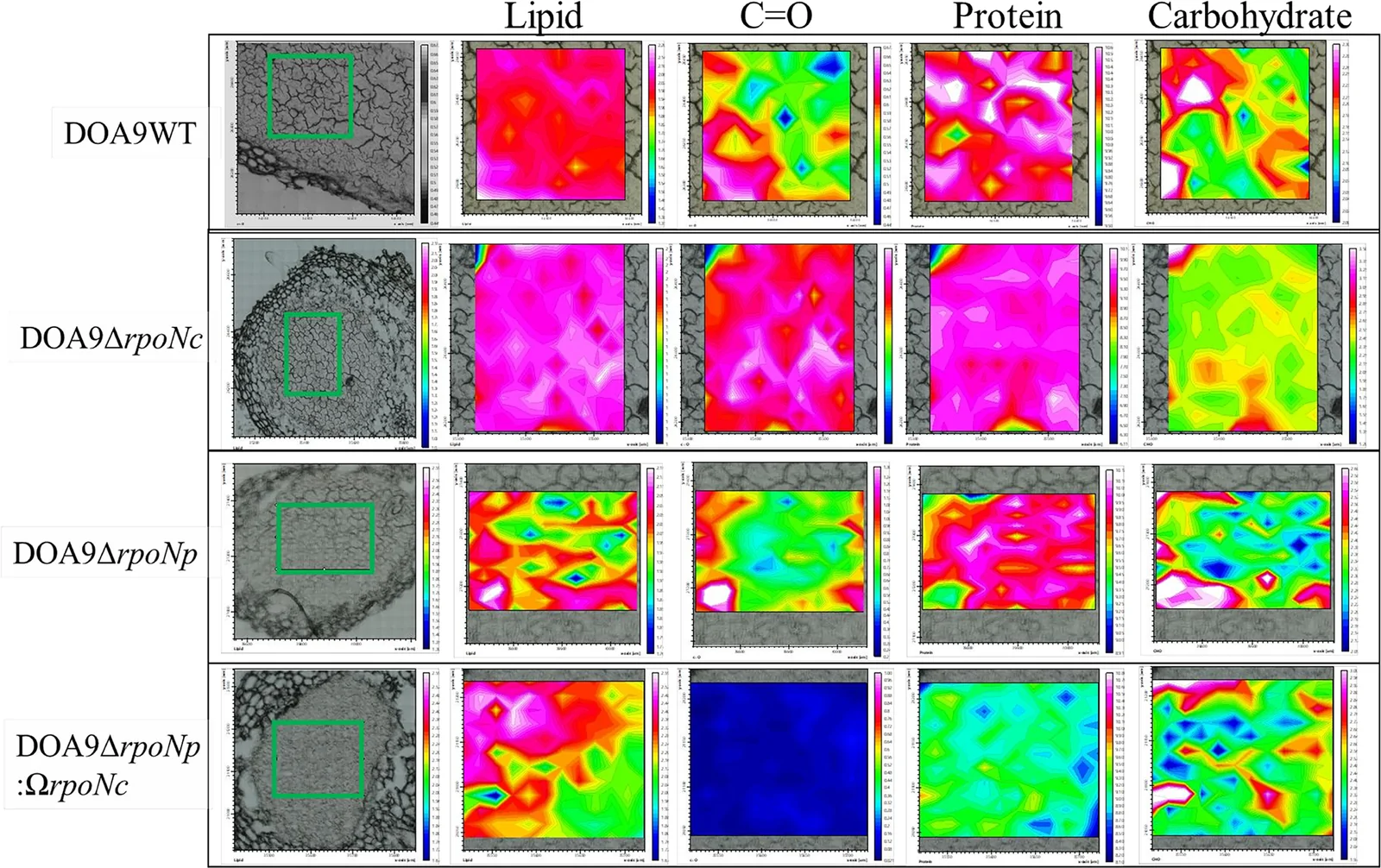
FTIR chemical mapping of cross-sectioned nodules. The map was used to enhance its visualization, with high amounts of chemical molecules colored pink and low amounts of chemical molecules colored blue. The measurement was performed by point-to-point mapping with aperture setting at 20 × 20  µm square aperture, 4 cm^−1^ 64 scans using SR-IR microspectroscopy over the area of dried nodule samples of *A. americana* after 20 days of inoculation with *Bradyrhizobium* sp. DOA9WT, DOA9∆*rpoNc*, DOA9∆*rpoNp,* and DOA9∆*rpoNp*:Ω*rpoNc*.

Nodules formed on *A. americana* derived by DOA9WT and its mutants displayed different phenotypes (Fig. S3). To investigate the biochemical composition of such nodules, FTIR microspectroscopy was applied. The FTIR feature of the symbiosomes exhibited information on complex biomolecules, such as bacteroids, proteins, enzymes, carbohydrates, sugars, amino acids, and minerals from the plant host. The average FTIR spectra of all strains showed some different levels of chemical profile (Fig. 4). To generate a chemical distribution map of the cross-sectioned nodule (Fig. 5), the area under the assigned chemical molecules was integrated, including lipid (3,000–2,800 cm^−1^), C=O (1,770–1,710 cm^−1^), protein (1,711–1,480 cm^−1^), and carbohydrate (1,152–956 cm^−1^). The false color in the map was used to enhance its visualization, with a large proportion of molecules colored pink and a few chemical molecules colored blue. The effective large nodule was obtained from DOA9WT nodulation, while the mutant strains induced a small and less effective phenotype. The chemical distribution map of the DOA9WT nodule had high homogeneity across the nodule, with a high concentration of lipid, protein, and carbohydrate. While an average of low homogeneity was observed in the DOA9∆*rpoNp* nodule, suggesting similar chemical mapping to the DOA9WT nodule. The DOA9∆*rpoNp*:Ω*rpoNc* nodule exhibited a significantly lower concentration of C=O than other strains (Fig. 5).

The protein band intensity of the DOA9∆*rpoNp*:Ω*rpoNc* nodule (Fig. 4A) was the lowest, indicating that the bacteroid concentration was lower than those of the other strains. This observation is consistent with the lower intensity of the C=O band, which corresponds to the phospholipid head group of the bacteroid membrane. The double mutation (DOA9∆*rpoNp*:Ω*rpoNc*) also had a significant impact on the CSP structure, as evidenced by a broader peak in the C–O–C region of the polysaccharide-pyranose ring and the absence of peaks at 1,304 cm^−1^ and 1,186 cm^−1^, which are responsible for the carbohydrate side chain. This finding provides insight into the mechanism behind why the double mutation causes a severe effect on nodulation.

The PCA analysis clearly classified nodules into four distinct groups (Fig. 4B). The double mutation (DOA9∆*rpoNp*:Ω*rpoNc*) was positioned far from the other groups. This data set was processed using the second derivative with ninth point smooth, causing the PC loading plot to be read in the opposite value. The negative PC1 (which represented 61% of the scores) separated the double mutation nodule from the other strains, as indicated by the negative values in the PC1 loading plot of the lipid group (2,915 cm^−1^ and 2,850 cm^−1^) and the protein bands (1,714 cm^−1^ and 1,623 cm^−1^) (Fig. 4C). These findings support the results of the average FTIR spectra.

### Elemental analysis of plant nodules using SEM-EDS

The SEM photograph of a sectioned nodule from a plant inoculated with different tested bacterial strains showed the different sizes and surfaces of sectioned nodules (see Fig. S4). Smaller nodules induced by *rpoN* mutants were observed compared to the sectioned nodule of DOA9WT (Fig. S4A through D). Based on the most similar plant phenotype and the remaining nitrogen fixation activity of small nodule samples from DOA9∆*rpoNp* compared to DOA9WT, the same nodule surface was observed in Fig. S4E and G by SEM photograph. In contrast, the small nodule phenotype from plants inoculated with DOA9∆*rpoNc* and DOA9∆*rpoNp*:Ω*rpoNc* strains showed a similar flake surface of sectioned nodules when compared to the massive surface of nodules from DOA9WT and DOA9∆*rpoNp* strains (Fig. S4F and H).

Interestingly, the chemical elements varied in plant nodule phenotypes using SEM-X-ray analysis, showing differences among the nodules (see Fig. S4; Table S1). The mass fraction of each species was indicated by the weight percent (%wt) of each detected chemical element, calculated as the species’ mass divided by the total mass of all species. The data showed that the %wt of the map spectrum of the small nodule phenotype collected from plants inoculated with all *rpoN* mutants decreased in O, P, K, S, and Al, while C increased as compared to DOA9WT. In addition, the effective nodules by DOA9WT and DOA9∆*rpoNp* were found to have a similar %wt of the map spectrum for Mg and O (Table S1). The reduction of %wt of the map spectrum of P, K, S, and Al found in small nodules of all *rpoN* mutants was related to their phenotype compared to the normal nodule by DOA9WT.

### Fe K-edge of XANES and EXAFS analysis of plant nodule

Leghemoglobin has been extensively studied in plant nodules and is known to have a significant impact on binding and transporting oxygen to the nitrogenase enzyme inside nodules. The nitrogenase enzyme converts atmospheric nitrogen (N_2_) into usable forms such as ammonia (NH_3_). Leghemoglobin contains the Fe–porphyrin ring, which is crucial in transporting oxygen. On the other hand, the nitrogenase enzyme requires the Fe–Mo cofactor (FeMo-co) located in the enzyme’s active site. To investigate the structure of the iron (Fe) complex in biological material within *A. americana* nodules nodulated by DOA9WT and its mutants, X-ray absorption spectroscopy (XANES and EXAFS) was used. XANES provides information on the electronic structure of materials by measuring the absorption of X-rays at the absorption edge of a Fe atom, while EXAFS provides information on the Fe local structure, including the distances between the Fe atom and its neighboring atoms, as well as the types of atoms surrounding the Fe atom.

**Fig 6:**
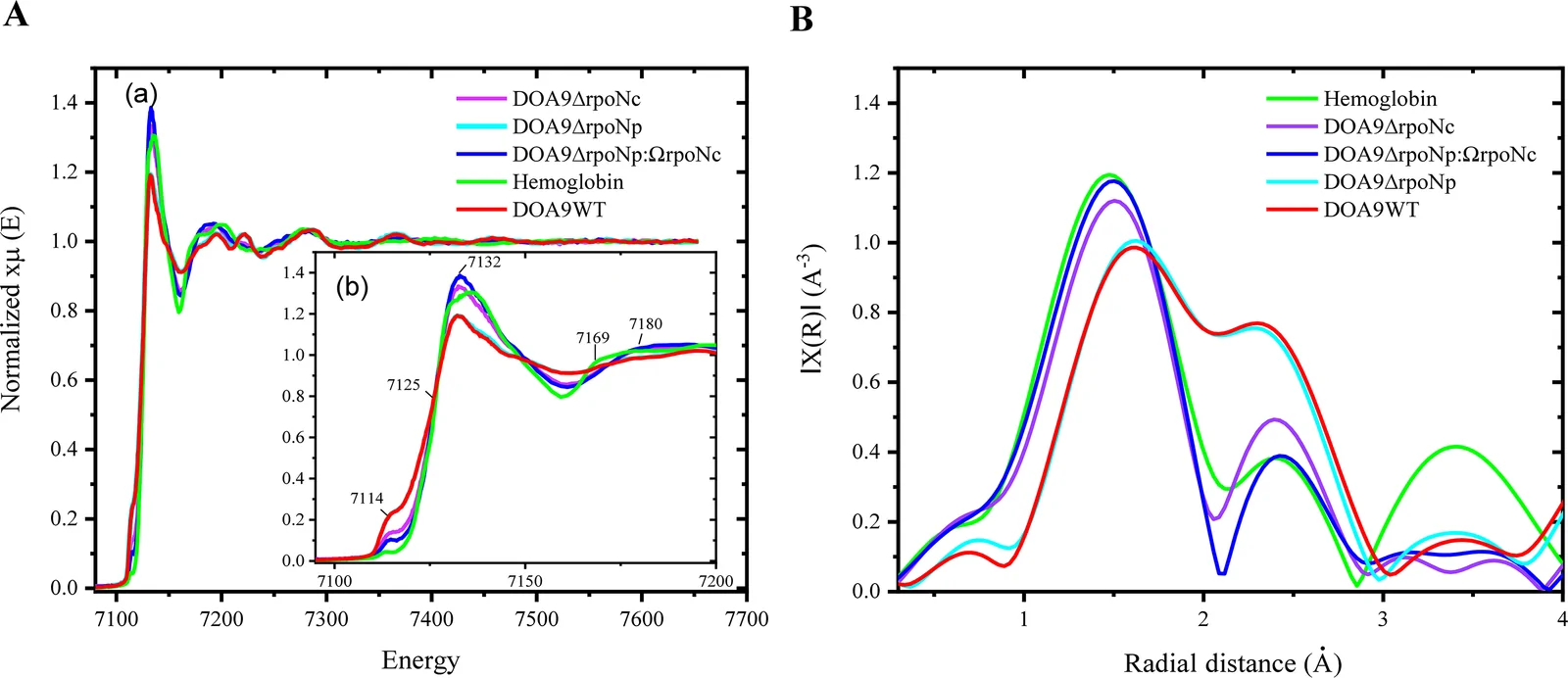
Analysis of nodule samples from the plants that were inoculated with DOA9WT (red line), DOA9∆*rpoNc* (purple line), DOA9∆*rpoNp* (blue line), and DOA9∆*rpoNp*:Ω*rpoNc* (dark blue line) compared to the standard spectra of hemoglobin (green line). The X-ray absorption spectroscopy (**A**) including the average Fe K-edge EXAFS spectra (a) and Fe K-edge XANES region (b). Fourier transforms of the Fe K-edge EXAFS (**B**).

All samples exhibited a Fe K-edge X-ray absorption near-edge structure (XANES) at 7125 eV, corresponding to Fe^3+^. The Fe K-edge XANES feature of DOA9WT was similar to that of DOA9∆*rpoNp* but significantly different from those of reference hemoglobin, DOA9∆*rpoNc* and DOA9∆*rpoNp*:Ω*rpoNc* (Fig. 6A). These results suggest that the Fe in DOA9WT and DOA9∆*rpoNp* has a different local environment compared to the Fe in the other samples. DOA9WT and DOA9∆*rpoNp* display the most intense 1s → 3d pre-edge transition, while hemoglobin exhibits the lowest. It is well-established that the pre-edge feature is typically transition forbidden but can exhibit increased intensity when the Fe center deviates from octahedral symmetry (30). Therefore, the higher intensity of the pre-edge feature observed in DOA9WT and DOA9∆*rpoNp* nodules suggests a greater presence of non-octahedral Fe biological material.

The Fe K-edge EXAFS spectra from all the nodule samples were Fourier transformed (FT) into *R* space to reveal structural details of the Fe coordination environment, including the distances between the Fe atom and its neighboring atoms (Fig. 6B). When the *R* space of all samples was compared to that of the reference hemoglobin, it was observed that DOA9∆*rpoNc* and DOA9∆*rpoNp*:Ω*rpoNc* exhibited similarities. Thus, the Fe–porphyrin ring of heme (31) was used for EXAFS fitting of such samples. On the other hand, DOA9WT and DOA9∆*rpoNp* samples were found to align at comparable positions but were different from hemoglobin (Fig. 6B). Therefore, the FeMo-co (32) was combined with the Fe–porphyrin ring for EXAFS fitting for these samples. The EXAFS best-fit parameters of all nodules are listed in Table 1.

**TABLE 1:** The EXAFS best-fit parameters of nodule samples

Fitting with Fe–porphyrin ring											
Fit parameter	*N*	WT		DOA9∆*rpoNp*		DOA9∆*rpoNc*		DOA9∆*rpoNp*:Ω*rpoNc*		Hemoglobin	
		*R*(Å)	*σ* ^2^	*R*(Å)	σ^2^	*R*(Å)	*σ* ^2^	*R*(Å)	σ^2^	*R*(Å)	*σ* ^2^
Fe–O	1	1.90331	0.00313	1.90431	0.00186	1.90798	0.00661	1.90524	0.00094	1.91155	0.00001
Fe–N	4	2.00528	0.00310	2.03289	0.00374	2.01986	0.00058	2.02579	0.00185	2.00567	0.00100
Fe–N	1	2.19791	0.00305	2.35880	0.00313	2.15010	0.00352	2.15010	0.00192	2.18688	0.00077
Fe–O–O	2	2.65752	0.00299	2.66892	0.00074	2.83902	0.00273	3.00746	0.00273	2.75448	0.00421
Fe–C	8	2.90798	0.00298	2.90542	0.00340	2.93425	0.00258	2.93157	0.00248	2.97261	0.00290
*R* factor		0.0113508		0.0037282		0.0104522		0.0165457		0.0288737	
SO^2^		0.591		0.595		0.91718		1.036		1.088	
∆E		−3.132		−1.991		3.933		2.427		1.686	

Fitting with Fe–Mo cofactor of nitrogenase enzyme											
Fit parameter	*N*	WT		DOA9∆*rpoNp*		DOA9∆*rpoNc*		DOA9∆*rpoNp*:Ω*rpoNc*		Hemoglobin	
		*R*(Å)	*σ* ^2^	*R*(Å)	*σ* ^2^	*R*(Å)	*σ* ^2^	*R*(Å)	*σ* ^2^	*R*(Å)	*σ* ^2^
Fe–S	2	2.25538	0.00310	2.25495	0.00201	–	–	–	–	–	–
Fe–S	1	2.25505	0.00291	2.25505	0.00298	–	–	–	–	–	–
Fe–S	1	2.25453	0.00313	2.25515	0.00297	–	–	–	–	–	–
Fe–Fe	1	2.51096	0.00290	2.50324	0.00312	–	–	–	–	–	–
Fe–Fe	1	2.89849	0.00701	2.87936	0.01174	–	–	–	–	–	–
Fe–Mo	1	2.80236	0.00302	2.88463	0.00189	–	–	–	–	–	–
SO^2^		0.409		0.405		–		–		–	
∆E		7.266		7.042		–		–		–	

**Fig 7:**
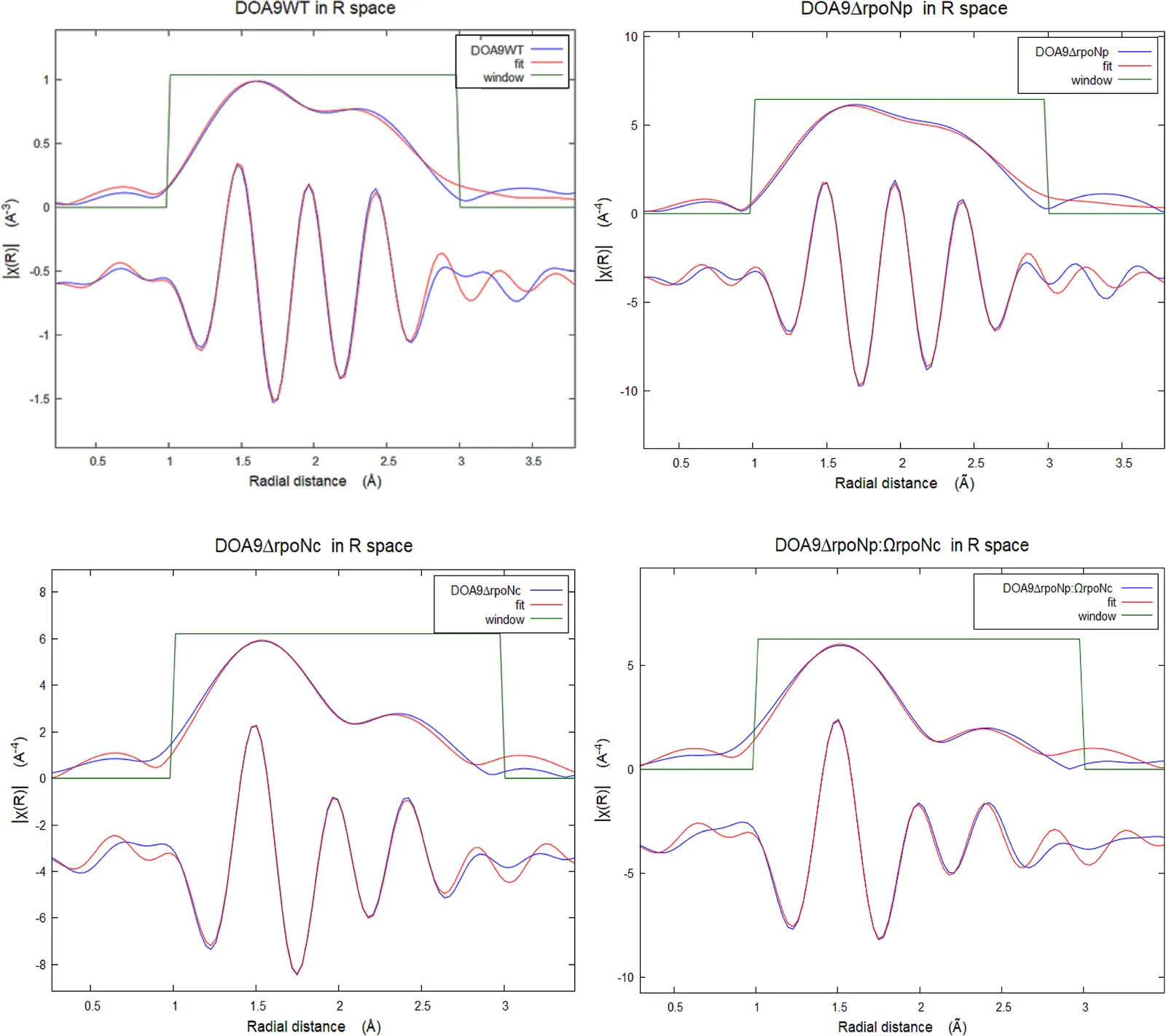
FT-EXAFS fitting of the nodule samples from the plants that were inoculated with DOA9WT, DOA9∆*rpoNc*, DOA9∆*rpoNp,* and DOA9∆*rpoNp*:Ω*rpoNc* after 20 days of cultivation compared to the reference hemoglobin structure.

The FT-EXAFS fit of the reference hemoglobin revealed the presence of five nitrogen neighbors around the Fe atom (Fe–N), with four similar distances and one different distance (~2.0–2.3 Å). The Fe–O bond was also identified, corresponding to the oxygen neighboring atom (~1.9 Å). The Fe–N and Fe–O distances observed in all the samples were similar (Table 1), indicating a consistent porphyrin ring composition. This suggests that leghemoglobin is the main component in the nodules. On the other hand, a sequential approach was used in fitting the FT-EXAFS of DOA9WT and DOA9∆*rpoNp* nodules. The approach first involves fixing all path parameters of hemoglobin achieved from the DOA9∆*rpoNc* sample and then adding definite path parameters of the Fe–Mo-co nitrogenase enzyme. Finally, the relative amplitude reduction of the porphyrin ring and Fe–Mo-co structure is determined. A good-fitting curve is shown in Fig. 7. The fitting results indicated that these nodules contain 59% leghemoglobin and 40% nitrogenase enzyme, as indicated by the value of amplitude reduction (SO^2^) (Table 1). The bond distances of Fe–Fe and Fe–Mo of the nitrogenase enzyme are longer than Fe–O and Fe–N in the Fe–porphyrin ring; thus, the *R* space of DOA9WT and DOA9∆*rpoNp* nodules shows different features than other mutants. This finding supports the XANES results, which showed that the pre-edge and spectral features of the Fe-XANES of the wild-type nodule are distinct from those of hemoglobin. Interestingly, mutations of the *rpoNp* gene on the plasmid have a smaller effect on nitrogenase production than mutations on the chromosome or the double mutation of both sides.

## DISCUSSION

Specific interactions during symbiotic nodulation and nitrogen fixation can improve the compatibility between rhizobia and leguminous plants. Various specific factors, including EPS, LPS, KPS, β-glucan, flagellins, and other secretion factors, have been investigated for their effects on nodulation efficiency and symbiotic nitrogen fixation (33, 34). In this symbiosis, nutrient elements also play an important role in plants and bacteria. This includes the synthesis of leghemoglobin for bacterial nitrogenase during nitrogen fixation and providing carbohydrate nutrition and energy for plant growth (2, 33). The expression of symbiosis-related genes in the early infection of specific rhizobia is significant for interacting with its host leguminous plant. In the previous work, we demonstrated that the regulatory RpoN is required to express other nitrogen-fixing genes under free-living and symbiotic conditions. Moreover, the study on the deletion of *rpoN* genes in DOA9 suggested an alternative sigma factor affecting colony morphology characteristics, leading to differences in its performance in nitrogen-fixing nodule phenotypes, as described by Wongdee et al. (13).

To our knowledge, this is the first study to combine FTIR and XAS techniques to report on the role of two RpoN proteins in regulating cellular surface polysaccharide (CSP) production and nitrogen-fixing root nodule phenotypes (Fix^−^ and Fix^+^) in *Bradyrhizobium* sp. strain. This study differs from traditional methods that rely on bacterial growth morphology and *in planta* experiments (13, 35, 36). Our findings revealed that *Bradyrhizobium* sp. could produce the CSP compound in liquid and solid media. Additionally, FTIR analysis has uncovered that the complexity of CSP production is greater in solid media than in liquid media (Fig. 3A). This suggests that the structural complexity of CSP, which is attached to the bacterial cells and secreted into the environment, may depend on specific nutritional supplements and growth conditions (37, 38). For the other aspect, quorum sensing may play an important role in different CSP production between culture media, as bacterial cells in solid media are more densely populated than those in liquid media, allowing for specific induction of bacterial communication based on population density. N-acyl homoserine lactones (AHLs), quorum-sensing molecules, have been reported to trigger the genes that regulate exopolysaccharide production in rhizobia. This leads to the formation of a biofilm and the development of a symbiotic relationship with the plant host (39).

The structure of CSP produced from DOA9WT is shown in Fig. 3, which exhibits a high intensity of the hydroxyl (–OH) group, the carboxylic acid (–COOH) group, polysaccharides-pyranose ring, and β-galactopyranosyl residues (at a wavenumber of 930, 889, and 874 cm^−1^). On the other hand, the *rpoN* mutant strains demonstrated low levels of these biomolecules, indicating that the mutation in *rpoN* led to a decrease in the production of lipids, galactose, galacturonic acid sugar, and poly-glucans in LPS and EPS structures. This finding is consistent with previously reported studies (4, 40, 41). The presence of galacturonic acid within the EPS structure of the *Bradyrhizobium* sp. strain plays a significant role in nodulation competitiveness for an indeterminate nodule on soybean roots (42). Moreover, bacterial biofilm formation on plant roots increases the nodulation ability, and the process of nodule maturation depends on bacterial galactose within the EPS structure (4, 43). Furthermore, poly-glucans, mostly called cyclic-glucans, are major cellular-saccharide envelope constituents of all members of the Rhizobiaceae family. The mutation at these loci, *ndv* and *chv* genes of *Rhizobium* sp., suggests the possible role of cyclic-glucans in bacterial growth in hypo-osmotic media and their ability to infect host plants (43, 44).

Both colony-CSP and liquid-CSP showed a high intensity of lipidic components in the wild-type strain, whereas the *rpoN* mutant strain showed a low intensity of these components (Fig. 3). This suggests that the structure of the lipopolysaccharide, which attaches to the membrane by a lipidic part inserted into the bacterial phospholipid layer [as explained by Carlson et al. (40)], was altered in the mutant strains. The general structure of LPS consists of an anchor named lipid A, which is associated with a core polysaccharide that can be linked to an O-antigen domain (40, 45). Defecting the synthesis of LPS in *R. trifolii* (40) and *Rhizobium* sp. NGR234 (42) impaired infection thread development and bacteroid differentiation, producing non-fixing root nodules. Similarly, symbiotic defects are observed when LPS production is defective in *R. leguminosarum* RBL5523, as is the case when the bacterium lacks the O-chain polysaccharide (OPS) (43, 44). The photosynthetic *Bradyrhizobium* strain, a symbiont of *Aeschynomene* legumes, synthesizes a unique LPS bearing a hopanoid-lipid A, which a hopanoid-deficient strain displays increased sensitivity to stressful conditions and a reduced ability to survive intracellularly in *A. evenia* (46). However, no significant effects on symbiotic nodulation were observed in other *Aeschynomene* species, such as *A. indica* and *A. afraspera* (47). The structural diversity of the O-antigen region is supposed to modulate or suppress plant defense responses to facilitate rhizobial symbiont establishment (36, 41, 45). Together, our results suggest that the differences in the structural components of CSP between DOA9WT and *rpoN* mutants, as analyzed by FTIR, may be the key factors involved in the symbiotic efficiency of DOA9WT with the host plant *A. americana*.

The FTIR measurements reveal that the double *rpoN* mutants, responsible for ineffective nitrogen-fixing nodules, display a lower concentration of biochemical molecules that are also more distinct compared to the effective nitrogen-fixing nodules induced by DO9WT (Fig. 4). In an *in vitro* experiment conducted without nitrogen supplementation, the presence of a healthy plant phenotype indicated that the bacteroids within the root nodules successfully generated functional nitrogenase (2, 13). According to Yu et al. (24), effective nitrogen-fixing nodules are characterized by high-frequency FTIR spectra of carbohydrates, proteins, lipids, and cellulose, indicative of plant growth and tissue development. SEM-EDS analysis provided supporting evidence, indicating that the nitrogen-fixing nodules of DOA9WT exhibited a high accumulation of biochemical elements, including O, P, and S, compared to the non-nitrogen-fixing nodules of DOA9∆*rpoNp*:Ω*rpoNc* (Fig. S4; Table S1). Furthermore, the higher amount of sulfur found in the nodules of DOA9WT is associated with a greater concentration of the Fe-Mo nitrogenase cofactor, leading to increased nitrogen fixation activity.

Analysis of the Fe-XANES pre-edge spectra revealed distinct features in the wild-type nodule and the plasmid mutant of the *rpoN* gene compared to reference hemoglobin (Fig. 6). Further FT-EXAFS fitting provided valuable insights into the composition of these nodules, indicating that they contain 59% leghemoglobin and 40% nitrogenase enzyme (Table 1). Notably, these findings suggest that mutations in the *rpoN* gene on the plasmid have a lesser impact on nitrogenase production than mutations on the chromosome or a double mutation in both areas. The impact of a mutation in the *rpoN* gene of *Bradyrhizobium* sp. strain on the phenotypic characteristics of plant nodules and nitrogen fixation efficiency has been previously reported (13).

### Conclusion

This study has demonstrated the crucial role of symbiont-produced CSPs in determining host plant specificity and effective nitrogen fixation. We used FTIR and XAS to investigate the structural changes in the CSPs and their impact on the biochemical composition of *Bradyrhizobium* sp. DOA9 and its *rpoN* mutants’ root nodule symbiosome components. Our findings indicate that mutations in the *rpoN* gene on the chromosome have a more pronounced effect on CSPs and nitrogenase production compared with *rpoN* mutations on the plasmid. The integrated use of synchrotron FTIR and XAS techniques in this research sheds light on the chemical characteristics of biological samples and provides a multidimensional perspective that can enhance our understanding of the complex processes involved in nitrogen fixation in root nodules. The application of these advanced spectroscopic techniques represents a significant contribution to the field of nitrogen fixation and emphasizes the importance of exploring innovative methodologies to expand our knowledge in this research area.

## MATERIALS AND METHODS

### Bacterial strains and culture condition

DOA9 WT, single rpoN mutants (∆rpoNc and ∆rpoNp), and a double mutant, ∆rpoNp:ΩrpoNc strains (13), were obtained from the Applied Soil Microbiology Laboratory, School of Biotechnology, Suranaree University of Technology, Thailand. These bacterial strains were cultured on YEM agar plates and in liquid YEM for 5 days at 28°C. To optimize the culturing conditions, appropriate antibiotics were added at the following concentrations: 300 µg/mL kanamycin and 20 µg/mL cefotaxime. The bacterial colonies on YEM agar plates were observed under *a microscope to assess their* morphologies.

### CSP extraction and preparation

To prepare the CSP production by DOA9 and all *rpoN* mutant strains, both YEM agar and liquid medium were used in this experiment. A single colony from a YEM plate that had been incubated for 5 days was selected and used to re-streak onto a new YEM agar plate. That resulted in the preparation of 20 plates used for colony-CSP extraction. After 7 days of cultivation, the cells were collected and resuspended in 20 mL of sterilized normal saline. The mixture was vigorously vortexed to dissolve the cells. The polysaccharide-cell suspension mixture was then centrifuged at 4,000 *g* for 5 minutes, and the CSP suspension was collected. This process was repeated five times to separate and precipitate the bacterial cells from the CSP suspension. The resulting extracted CSPs were termed colony-CSP.

To extract the CSPs from the liquid cultures (referred to as liquid-CSP), 1 L of liquid YEM medium with a 1% (wt/wt) inoculation was used. After a 5-day incubation period, the bacterial cultures were centrifuged at 8,000 *g* for 15 minutes using a tabletop centrifuge, and the cell pellets were discarded. The tubes containing the polysaccharide suspension from colony-CSP and the collected supernatant containing liquid-CSP were maintained at 4°C, and ice-cold ethanol [95% (vol/vol)] was added until reaching a final concentration of 35% (vol/vol). The mixture was then vigorously mixed in a multi-vortex mixer and incubated overnight at 4°C. The precipitated CSP was centrifuged at 8,000 *g* for 20 minutes at 4°C. Any residual ethanol was then evaporated in a laminar flow hood for 5 hours. Subsequently, the CSP was subjected to lyophilization. The weight of the resulting lyophilized CSP pellet was measured in milligrams (mg) of dried weight and used for further analyses. This protocol was modified based on the procedure of Bomfeti et al. and Castellane et al. (4, 5). To investigate the pellet features and microstructures of dried colony-CSP and liquid-CSP, we used a FE-SEM. The CSP samples were prepared by dissolving 1 mg of dried powder in 1 mL of deionized water (DI). A mixed solution at a dilution of 10^−3^ was prepared, and 2 µL of this solution was placed on infrared transparent barium fluoride (BaF_2_) windows for FTIR analysis. These sample drops were completely dehydrated in a vacuum desiccator. The window containing sample drops was further subjected to SR-FTIR microspectroscopy.

### Legume nodule preparation

The nodules elicited by DOA9WT, DOA9∆*rpoNc*, DOA9∆*rpoNp*, and DOA9∆*rpoNp*:Ω*rpoNc* strains on *A. americana* were prepared following the protocol from Wongdee et al. (13). The collected nodules were cryo-embedded using optimal cutting temperature (OCT) according to the following procedure: the nodules were placed into the cryomold containing the OCT compound. The cryomold was then frozen using liquid nitrogen. Subsequently, the embedded nodules were thinly sectioned to 5–10 μm using an ultramicrotome (LEICA CM1950). Thin sections of the nodules were carefully selected and placed on a BaF_2_ window. The sections were then left to dry overnight or until completely dried in a vacuum desiccator. Once the sections were fully dried, the window containing the nodule sections was ready for FTIR analysis.

For SEM imaging, the fresh nodule was sliced into thin sections of 25 µm. The cross-sectioned nodules were then vertically mounted on a stub using carbon tape. Subsequently, the nodule sections underwent a 30-minute sputtering process with 99.9% gold (Neocoater/mp-19020NCTR) for optimal imaging.

For XAS analysis, the freshly collected nodules underwent a cleaning procedure involving five washes with sterilized water. Subsequently, the nodules were dried using lyophilization. The dried nodules were then finely ground into a powder to prepare them for XAS analysis. Finally, the powdered samples were placed into an XAS frame for subsequent experimentation.

### CSP and root nodule analysis using SR-FTIR microspectroscopy

FTIR measurement was conducted at BL4.1 IR Spectroscopy and Imaging at the Synchrotron Light Research Institute (Public Organization) (SLRI), Nakhon Ratchasima, Thailand. Spectra were acquired with a Vertex 70 FTIR spectrometer (Bruker Optics, Ettlingen, Germany), an IR microscope (Hyperion 2000, Bruker), and an MCT detector cooled with liquid nitrogen during the measurement range between 4,000 and 400 cm^−1^. The microscope was connected to a software-controlled microscope stage and placed in a specially designed box purged with dry air. The measurements were performed in mapping mode, using an aperture size of 20 × 20 µm with a spectral resolution of 4 cm^−1^, with 64 scans co-added. Spectral acquisition and instrument control were performed using OPUS 7.2 (Bruker Optics Ltd., Ettlingen, Germany) software.

The sample drops from both colony-CSP and liquid-CSP were measured, and an average spectrum was generated based on 50 spectra. The spectra were processed using a nine-point smoothing technique and normalization with extended multiplicative signal correction. This processing was performed over the spectral range of 3,800–900 cm^−1^. For the sectioned nodules, area measurements were made at the middle of each nodule. PCA was conducted using the Unscrambler X software (CAMO). Peak integration analysis was carried out for the assigned chemical molecules, including –OH (3,600–3,000 cm^−1^), CH_2_, CH_3_ of lipid (3,000–2,800 cm^−1^), C=O (1,770–1,710 cm^−1^), protein (1,711–1,480 cm^−1^), and C–O–C of carbohydrate (1,152–956 cm^−1^). The chemical distribution map was rendered using false colors to enhance visualization, with pink color indicating areas of high molecule concentration and blue color representing regions of lower concentration.

### Elemental analysis of plant nodules using SEM-EDS

The thin section of nodule samples was observed for tissue texture morphology using SEM on the FEI QUANTA 450 (ThermoFisher Scientific, USA) equipped with EDS (Oxford EDS Xmax). Images were taken in high-vacuum mode with a voltage of 20 kV.

### X-ray absorption spectroscopy measurements

XAS spectra were recorded at the SUT-NANOTEC-SLRI XAS beamline (BL5.2) of the SLRI, Thailand. The synchrotron light had an electron energy of 1.2 GeV with a beam current of 80–150 mA. The X-ray light was produced from a bending magnet to obtain the energy range of 1,240–12,100 eV and a photon flux ranging from 10^8^ to 10^10^ photons/s/100 mA. Energy calibration was performed using a Fe foil in transmission mode. To scan the Fe K-edge XANES spectra, a Ge (220) double crystal monochromator was used to scan from −20 eV to 80 eV of *E*_0_ at 7,112 eV, with a photon energy step of 0.2 eV. For EXAFS measurement, the photon energy scan was set up in a range of −150, –20, 30, and 13k of *E*_0_ at 7,112 eV with a photon energy step of 5, 0.3, 0.05k eV, and a time step of 1, 3, and 5 s. The samples were measured up to five scans in fluorescent mode using a 4-element Si-drift detector. The IFEFFIT program, which incorporates Athena and Artemis (48) was used to preprocess and normalize the XANES data. Hemoglobin from bovine blood [lyophilized powder (H2500; SIGMA-ALDRICH)] was used as the Fe-porphyrin ring reference material. Subsequently, the FT-EXAFS was fitted with the structure of the Fe-porphyrin ring of the PubChem CID 139127581 with modified N6 to O atom and Fe–Mo cofactor (COD ID 4113594) of the nitrogenase enzyme.

## SUPPLEMENTAL MATERIAL

Fig. S1 spectrum.01947-23-s0001.pdf The colony morphology of *Bradyrhizobium* sp. strain DOA9WT, DOA9∆rpoNc, DOA9∆rpoNp DOA9∆rpoNp:ΩrpoNc on solid YEM medium (A-D), the single colony was observed under microscopy (E-H). In A-D and E-H, the bars were indicated as 300 and 200 micrometer (µm), respectively.

Fig. S2 spectrum.01947-23-s0002.pdf The functional groups present in colony-CSP of different bacterial strains were analyzed for the percent integral area of each characteristic FTIR peaks including the functional groups of -OH, CH2 and CH3 (lipid), C=O, -C-O-C (carbohydrate), and -COOH (protein).

Fig. S3 spectrum.01947-23-s0003.pdf Different plant phenotype of *Aeschynomene americana* after inoculation with different *Bradyrhizobium* sp. strains; DOA9 wild-type (WT), DOA9ΔrpoNc, DOA9ΔrpoNp, and DOA9ΔrpoNp:ΩrpoNc strains (A).

Fig. S4 spectrum.01947-23-s0004.pdf SEM-EDS photograph analysis of nodule samples from the plants that inoculated with DOA9WT, DOA9∆rpoNc, DOA9∆rpoNp and DOA9∆rpoNp:ΩrpoNc after 20 days of cultivation. SEM photograph showed overall picture of the actual sample size of thin sectioned nodules (A-D). The fine photograph of each thin sectioned nodules was magnified and observed the tissue surfaces (E-H). The electron transmitting photograph of thin sectioned nodules were combined and observed for EDS level of %wt (I-L). The bars were indicated as 250 and 10 micrometers (µm) for SEM and EDS, respectively.

Table S1 spectrum.01947-23-s0005.docx The percent wt of map spectrum analyzed mass fraction of species in nodule sample of plant that elicited with different bradyrhizobial tested strains.

ASM does not own the copyrights to Supplemental Material that may be linked to, or accessed through, an article. The authors have granted ASM a non-exclusive, world-wide license to publish the Supplemental Material files. Please contact the corresponding author directly for reuse.
