# Supplementary material for: Exploring the cellular surface polysaccharide and root nodule symbiosis characteristics of the rpoN mutants of Bradyrhizobium sp. DOA9 using synchrotron-based Fourier transform infrared microspectroscopy in conjunction with X-ray absorption spectroscopy
Source: Microbiol Spectr. 2023 Sep 8;11(5):e01947-23. doi: 10.1128/spectrum.01947-23 (PMC10581086; doi:10.1128/spectrum.01947-23)
Supplement: Fig. S1 — The colony morphology of Bradyrhizobium sp. strain DOA9WT, DOA9∆rpoNc, DOA9∆rpoNp DOA9∆rpoNp:ΩrpoNc on solid YEM medium (A-D), the single colony was observed under microscopy (E-H). In A-D and E-H, the bars were indicated as 300 and 200 micrometer (µm), respectively. [file spectrum.01947-23-s0001.pdf]

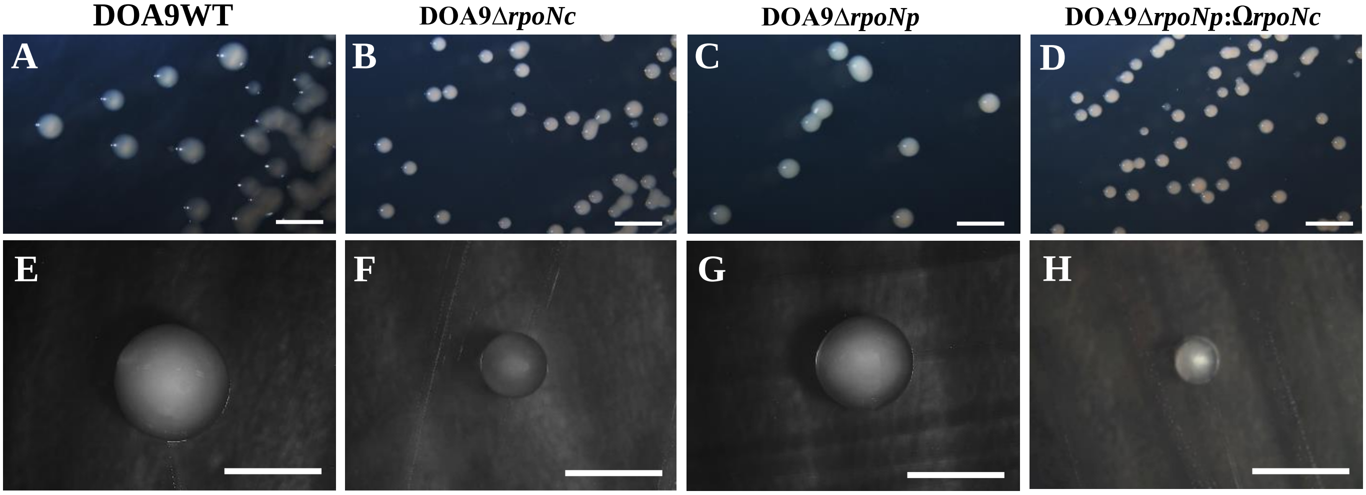

Figure S1. The colony morphology of *Bradyrhizobium* sp. strain DOA9WT, DOA9Δ*rpoNc*, DOA9Δ*rpoNp* DOA9Δ*rpoNp*:Ω*rpoNc* on solid YEM medium (**A-D**), the single colony was observed under microscopy (**E-H**). In **A**-**D** and **E**-**H**, the bars were indicated as 300 and 200 micrometer (μm), respectively.
