# Supplementary material for: Exploring the cellular surface polysaccharide and root nodule symbiosis characteristics of the rpoN mutants of Bradyrhizobium sp. DOA9 using synchrotron-based Fourier transform infrared microspectroscopy in conjunction with X-ray absorption spectroscopy
Source: Microbiol Spectr. 2023 Sep 8;11(5):e01947-23. doi: 10.1128/spectrum.01947-23 (PMC10581086; doi:10.1128/spectrum.01947-23)
Supplement: Fig. S2 — The functional groups present in colony-CSP of different bacterial strains were analyzed for the percent integral area of each characteristic FTIR peaks including the functional groups of -OH, CH2 and CH3 (lipid), C=O, -C-O-C (carbohydrate), and -COOH (protein). [file spectrum.01947-23-s0002.pdf]

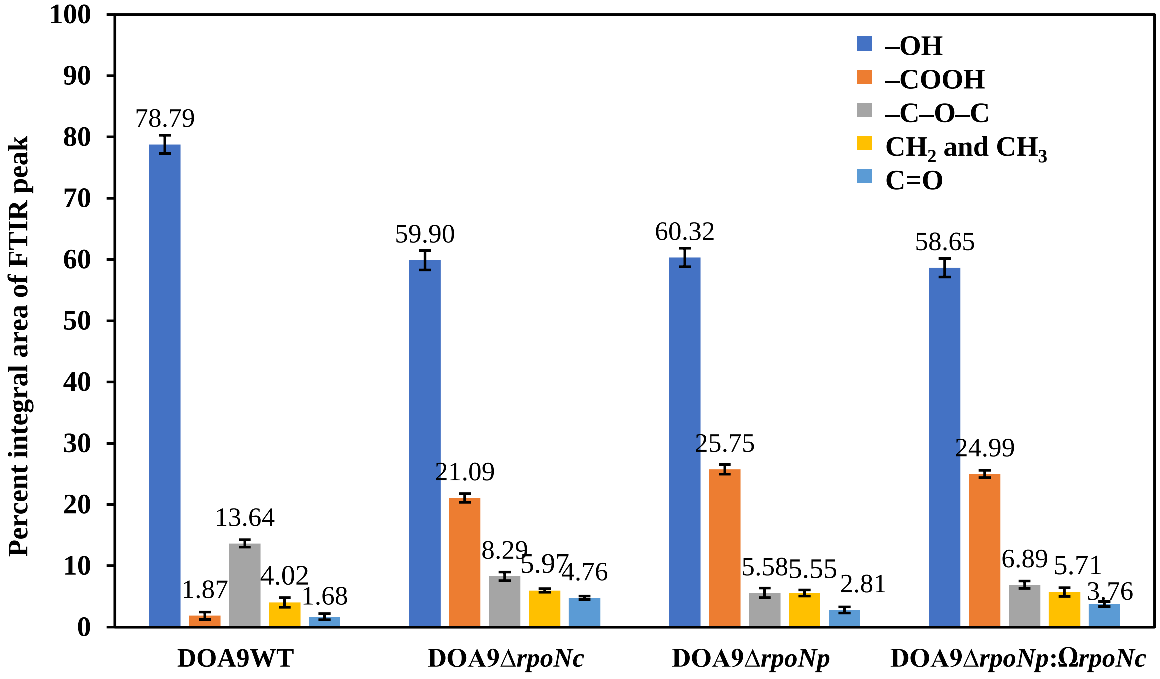

Figure S2. The functional groups present in colony-CSP of different bacterial strains were analyzed for the percent integral area of each characteristic FTIR peaks including the functional groups of –OH, CH_2 and CH_3 (lipid), C=O, –C–O–C (carbohydrate), and –COOH (protein).
