# Supplementary material for: Exploring the cellular surface polysaccharide and root nodule symbiosis characteristics of the rpoN mutants of Bradyrhizobium sp. DOA9 using synchrotron-based Fourier transform infrared microspectroscopy in conjunction with X-ray absorption spectroscopy
Source: Microbiol Spectr. 2023 Sep 8;11(5):e01947-23. doi: 10.1128/spectrum.01947-23 (PMC10581086; doi:10.1128/spectrum.01947-23)
Supplement: Fig. S3 — Different plant phenotype of Aeschynomene americana after inoculation with different Bradyrhizobium sp. strains; DOA9 wild-type (WT), DOA9ΔrpoNc, DOA9ΔrpoNp, and DOA9ΔrpoNp:ΩrpoNc strains (A). [file spectrum.01947-23-s0003.pdf]

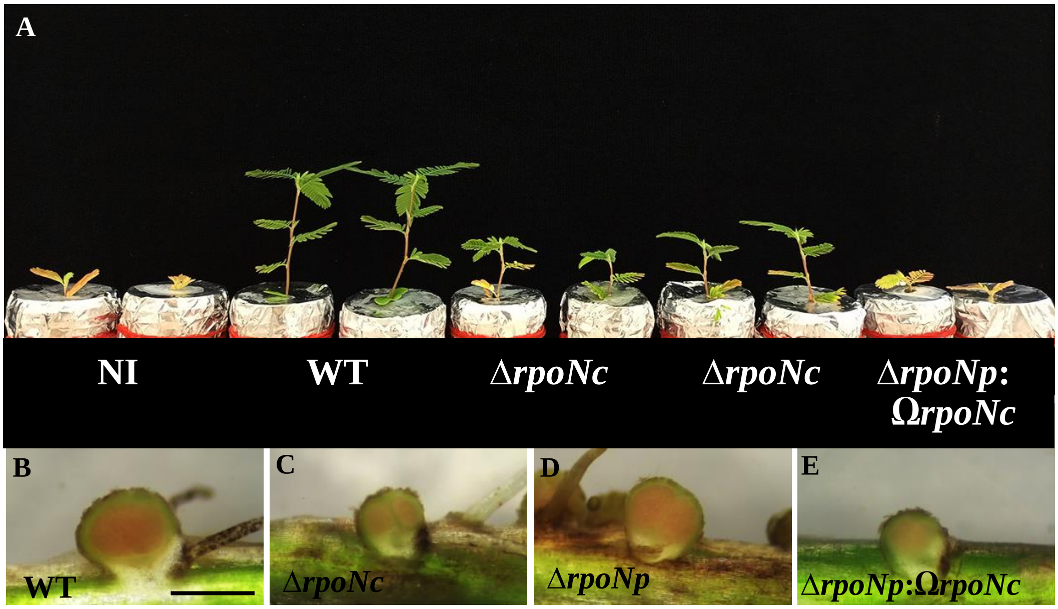

Figure S3. Different plant phenotype of *Aeschynomene americana* after inoculation with different *Bradyrhizobium* sp. strains; DOA9 wild-type (WT), DOA9Δ*rpoNc*, DOA9Δ*rpoNp*, and DOA9Δ*rpoNp*:Ω*rpoNc* strains (**A**).
