# Supplementary material for: Exploring the cellular surface polysaccharide and root nodule symbiosis characteristics of the rpoN mutants of Bradyrhizobium sp. DOA9 using synchrotron-based Fourier transform infrared microspectroscopy in conjunction with X-ray absorption spectroscopy
Source: Microbiol Spectr. 2023 Sep 8;11(5):e01947-23. doi: 10.1128/spectrum.01947-23 (PMC10581086; doi:10.1128/spectrum.01947-23)
Supplement: Fig. S4 — SEM-EDS photograph analysis of nodule samples from the plants that inoculated with DOA9WT, DOA9∆rpoNc, DOA9∆rpoNp and DOA9∆rpoNp:ΩrpoNc after 20 days of cultivation. SEM photograph showed overall picture of the actual sample size of thin sectioned nodules (A-D). The fine photograph of each thin sectioned nodules was magnified and observed the tissue surfaces (E-H). The electron transmitting photograph of thin sectioned nodules were combined and observed for EDS level of %wt (I-L). The bars were indicated as 250 and 10 micrometers (µm) for SEM and EDS, respectively. [file spectrum.01947-23-s0004.pdf]

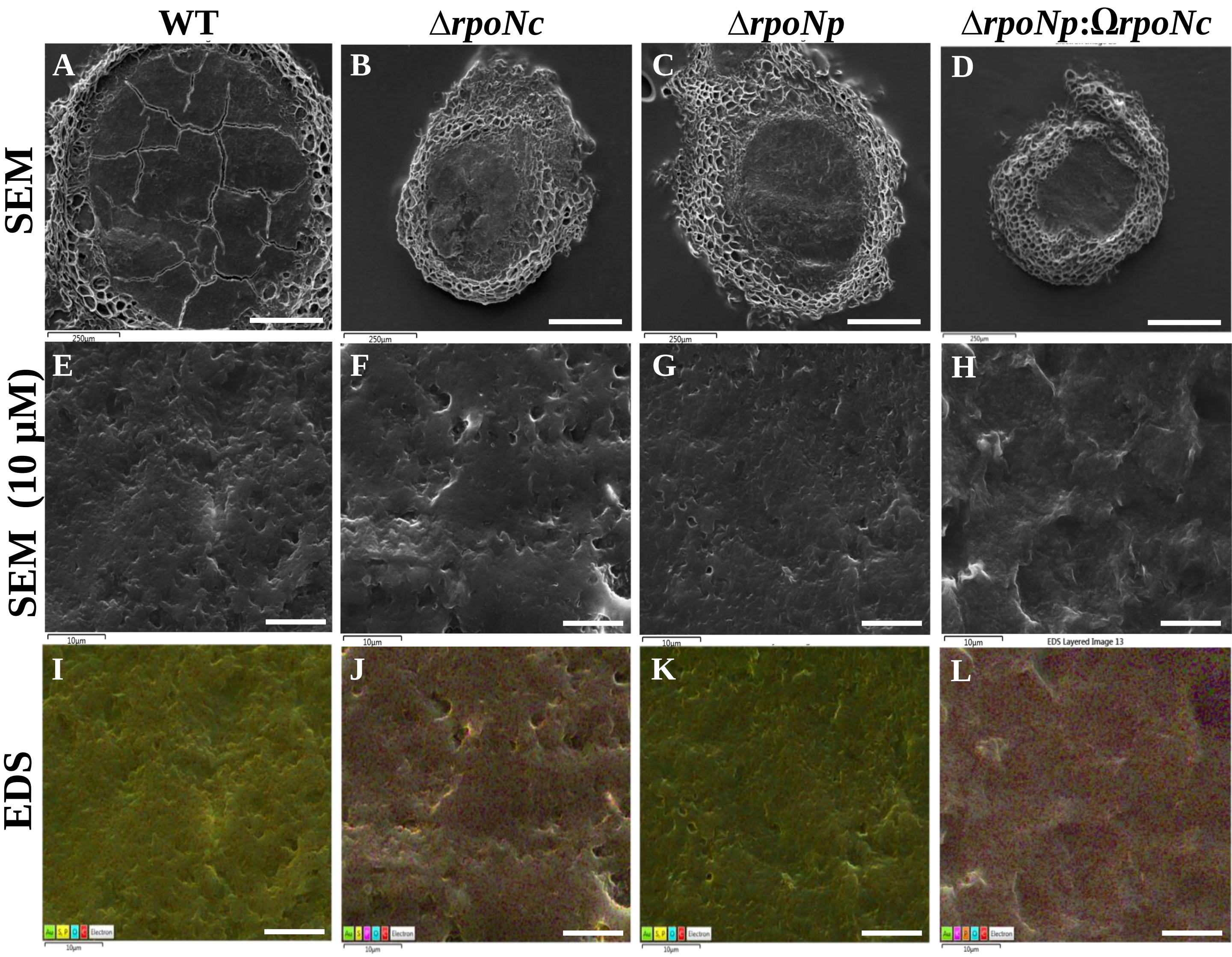

Figure S4. SEM-EDS photograph analysis of nodule samples from the plants that inoculated with DOA9WT, DOA9Δ*rpoNc*, DOA9Δ*rpoNp* and DOA9Δ*rpoNp*:Ω*rpoNc*. SEM photograph of thin sectioned nodules (**A**-**D**). The fine photograph of thin sectioned nodules was magnified the tissue surfaces (**E**-**H**). The electron transmitting photograph for EDS level of %wt (**I**-**L**). The bars were indicated as 250 and 10 micrometers (μm) for SEM and EDS, respectively.
