# Supplementary material for: Exploring the cellular surface polysaccharide and root nodule symbiosis characteristics of the rpoN mutants of Bradyrhizobium sp. DOA9 using synchrotron-based Fourier transform infrared microspectroscopy in conjunction with X-ray absorption spectroscopy
Source: Microbiol Spectr. 2023 Sep 8;11(5):e01947-23. doi: 10.1128/spectrum.01947-23 (PMC10581086; doi:10.1128/spectrum.01947-23)
Supplement: Table S1 — The percent wt of map spectrum analyzed mass fraction of species in nodule sample of plant that elicited with different bradyrhizobial tested strains. [file spectrum.01947-23-s0005.docx]

| Mass fraction of species | % wt of map spectrum analyzed in plant nodules^a^ | | | |
| --- | --- | --- | --- | --- |
|  | DOA9WT | ∆*rpoNc* | ∆*rpoNp* | ∆*rpoNp*:Ω*rpoNc* |
| C | 70.5±2.0a | 73.1±1.0a | 71.2±2.0a | 72.1±1.0a |
| O | 27.7±0.2a | 25.0±0.1a | 26.5±0.2a | 26.4±0.1a |
| P | 0.7±0.0a | 0.4±0.0c | 0.5±0.0b | 0.3±0.0d |
| K | 0.5±0.0a | 0.3±0.0b | 0.3±0.0b | 0.2±0.0c |
| S | 0.6±0.0a | 0.5±0.0b | 0.4±0.0c | 0.2±0.0d |
| Mg | 0.3±0.0a | 0.1±0.0c | 0.3±0.0a | 0.2±0.0b |
| Al | 0.2±0.0a | 0.1±0.0b | 0.1±0.0b | 0.1±0.0b |
| Cl | 0.1±0.0a | 0.1±0.0a | 0.1±0.0a | 0.1±0.0a |
| Ca | 0.2±0.0a | 0.2±0.0a | 0.2±0.0a | 0.2±0.0a |
| Na | 0.1±0.0a | 0.1±0.0a | 0.1±0.0a | 0.1±0.0a |
| Fe | 0.1±0.0a | 0.1±0.0a | 0.1±0.0a | - |

**Table S1.** The percent wt of map spectrum analyzed mass fraction of species in nodule sample of plant that elicited with different bradyrhizobial tested strains.

^a^ These data sets were represented the mean values of 3 replicates, and letters of statistically significant differences in the same row (ANOVA Tukey test (*p*<0.01)) were indicated.
